# Usefulness of broad-range PCR in the etiologic diagnosis of sepsis

**DOI:** 10.1186/cc12942

**Published:** 2013-11-05

**Authors:** Aline Gozzi, Romulo R Lobo, José Maurício SC Mota, Antonio Pazin Filho, Benedito AL Fonseca, Marcos C Borges

**Affiliations:** 1 Department of Internal Medicine, Ribeirão Preto Medical School, University of São Paulo, Ribeirão Preto, SP, Brazil

## Background

Sepsis is responsible for a high rate of hospitalization and mortality. Traditional methods for bacterial isolation and identification, such as blood culture, have limitations in sensitivity and specificity and their results usually are not available before 48 to 72 hours. The PCR allows a rapid diagnosis of infectious agents. Broad-range PCR allows an earlier and more sensitive bacterial identification in just one reaction, even after the initiation of antibiotics. Therefore, this study evaluates the use of broad-range PCR in the etiologic diagnosis of septic patients, and compares it with traditional methods of culture.

## Materials and methods

Thirty-five patients with sepsis, admitted to the Emergency Unit of Clinical Hospital of Ribeirao Preto Medical School, were included in the study. Clinical, laboratory, and culture data were collected at hospital admission. On the first day of admission, DNA extraction was performed from serum, plasma and buffy-coat samples from all patients. Broad-range 16S rDNA PCR was then performed using two different pairs of primers (Bak11W/Bak2 and Taf/Tar).

## Results

Eighteen (51%) patients were female; mean age was 58 ± 18 years; 15 (43%) had severe sepsis and 20 (57%) septic shock, and mortality was 54%. Mean C-reactive protein (CRP) was 14.39 ± 9.52 and 28 (80%) patients have levels of CRP greater than 5.0 ng/l. The primary site of infection was detected in all patients, 20 (57%) patients had respiratory tract infection, nine (26%) urogenital tract infection, three (8.5%) cutaneous infection, and three (8.5%) other sites. Blood culture was positive in 14 (40%) samples. Broad-range PCR was positive in 19 (51%) samples. Only 10 (29%) samples were positive for both techniques. In 11 (31%) patients, neither blood culture nor PCR were positive.

## Conclusions

Broad-range PCR was effective for diagnosis of bacterial infection in septic patients, and could be an option to be used in patients with severe sepsis and septic shock. Moreover, it is faster than blood culture and can detect bacteria even after the initiation of intravenous antibiotics. The combination of both techniques could increase the likelihood of etiologic diagnostic in septic patients.

